# Quality Assurance of Point and 2D Shear Wave Elastography through the Establishment of Baseline Data Using Phantoms

**DOI:** 10.3390/s24154961

**Published:** 2024-07-31

**Authors:** Jacqueline Gallet, Elisabetta Sassaroli, Qing Yuan, Areej Aljabal, Mi-Ae Park

**Affiliations:** 1 Department of Radiology, Division of Medical Physics, UT Southwestern Medical Center, Dallas, TX 75390, USA; qing.yuan@utsouthwestern.edu (Q.Y.); areej.aljabal@utsouthwestern.edu (A.A.); 2 STEM Department, MassBay Community College, Wellesley, MA 02481, USA; esassaroli@massbay.edu

**Keywords:** ultrasound elastography, quality assurance, elastography phantom, 2D sheer wave

## Abstract

Ultrasound elastography has been available on most modern systems; however, the implementation of quality processes tends to be ad hoc. It is essential for a medical physicist to benchmark elastography measurements on each system and track them over time, especially after major software upgrades or repairs. This study aims to establish baseline data using phantoms and monitor them for quality assurance in elastography. In this paper, we utilized two phantoms: a set of cylinders, each with a composite material with varying Young’s moduli, and an anthropomorphic abdominal phantom containing a liver modeled to represent early-stage fibrosis. These phantoms were imaged using three ultrasound manufacturers’ elastography functions with either point or 2D elastography. The abdominal phantom was also imaged using magnetic resonance elastography (MRE) as it is recognized as the non-invasive gold standard for staging liver fibrosis. The scaling factor was determined based on the data acquired using MR and US elastography from the same vendor. The ultrasound elastography measurements showed inconsistency between different manufacturers, but within the same manufacturer, the measurements showed high repeatability. In conclusion, we have established baseline data for quality assurance procedures and specified the criteria for the acceptable range in liver fibrosis phantoms during routine testing.

## 1. Introduction

Increased stiffness through palpation is routinely used in the screening of prostate, cervical, and breast cancers. However, its application is limited to accessible organs, and it is a purely subjective method with a limited ability to detect disease. In recent years, ultrasound elastography has become a widely available imaging technique aimed at overcoming these limitations. The two main clinical applications of ultrasound elastography to date have been for evaluating liver fibrosis or for differentiating malignant from benign tumors in various organs, such as the breast. For a general overview of ultrasound elastography, see for example [1,2,3,4]. In ultrasound elastography, remote palpation is implemented clinically by the application of a force to induce tissue deformation. Techniques available in clinical practice include strain elastography (SE), acoustic radiation force impulse (ARFI) imaging, transient elastography (TE), and shear wave elastography (SWE).

In SE [5,6], a quasi-static compressive force is applied manually to induce a tissue axial displacement, which is converted to strain (percent displacement) to generate a strain map of the tissue. ARFI imaging [7] images the displacement distribution generated by the acoustic radiation force. TE techniques [8,9] measure the shear wave speed (SWS) generated by an external vibrating source, a controlled vibrating thump at the tissue surface. SWE techniques [10,11,12,13] measure the shear wave speed (SWS) generated by the acoustic radiation force.

This paper focuses on the evaluation of two SWE modes: point-SWE (p-SWE) and 2D-SWE, both available on clinical ultrasound imaging systems. In p-SWE, a focused ultrasound pulse with a longer duration (several hundred microseconds) than the one typically used in diagnostic ultrasound imaging is focused near the region of interest (ROI) to induce tissue displacement on the order of a few micrometers in the direction of the US beam. When the transmission of the push pulse is completed, the displaced tissue returns to its original position and a spherical shape shear wave is generated, propagating mainly perpendicularly to the focused ultrasound beam’s axis. In soft tissue, shear waves are only generated at low frequencies (10 Hz to 2000 Hz) and propagate more slowly than compressional waves with a speed in the range 1–10 m/s. In this mode, statistical quantities such as the mean, the median, and standard deviation (SD) of the SWS in the ROI are sometimes provided by the manufacturer.

In the push-pulse transmission with a single focal point, the region in which the SWS can be measured is usually narrow due to the large attenuation of shear waves. In 2D-SWE, an image of the SWS distribution in a larger ROI is provided. This is accomplished by placing the push pulse at multiple sequential locations and, at each, detecting the shear wave arrival time at multiple lateral locations. This creates patches of small SWS images that are used to create a large ROI 2D-SWE image, which can be displayed in color against a color scale, calibrated in m/s or kPa. Quantitative measurements can be obtained by direct measurements within the ROIs (circles or boxes). These ROIs can be placed at desired locations to obtain statistical quantities such as the mean, median SWS, or Young’s modulus. No color is displayed in a confidence map if the signal-to-noise ratio is not considered adequate by the system software for all manufacturers offering some form of ultrasound elastography.

All the current elastography techniques assume soft tissue to be a linearly elastic, homogeneous, isotropic, infinite, and incompressible medium. For such a medium, SWS is constant and related to the tissue shear modulus (G) and Young’s modulus (elastic modulus) by

(1) $$SWS=\sqrt{\frac{G}{\rho }}=\sqrt{\frac{E}{3\rho }}$$

with ρ being the mass density of soft tissue and E=3G. Young’s modulus (E), defined as the ratio between the applied stress and the resulting strain (Hooke’s law), E=stress/strain, is generally used as a physical quantity to quantify tissue stiffness.

SWS measurements are susceptible to biases. These biases are inevitable and are caused by the shear wave not behaving as the ultrasound imaging system assumes. In [14], the factors influencing the accuracy of SWS measurements were classified as physical factors and engineering factors. Physical factors include the phenomena associated with the shear wave propagation of and interaction with tissue and the assumed mechanical properties of tissue. Engineering factors include the ones associated with the image system performance (for example, spatial resolution, image contrast, etc.) and the techniques developed for the generation and detection of the shear waves.

As longitudinal waves and also shear waves propagating in tissue undergo reflection, refraction, interference, diffraction, and attenuation, these physical factors may cause artifacts. In addition, a shear wave cannot propagate in liquids. Equation (1) is valid for a linear, elastic, and isotropic medium. However, in general, soft tissues show viscoelasticity, nonlinear elasticity, and anisotropy. These properties affect the evaluation of a tissue’s stiffness in a complex way. Viscoelasticity makes the phase velocity and the shear modulus frequency dependent [15,16,17]. Nonlinearity makes the SWS and shear modulus dependent on the magnitude of applied strain or stress [18,19]. Tissues, for example, muscle fibers, show anisotropy, where the elastic modulus depends on the direction. The existence of anisotropy, where SWSs differ depending on the direction of shear wave propagation, has been reported [20,21]. In tissue, the wavelength of a shear wave is on the order of a few mm. Therefore, it is expected that when the size of the lesion (for example, a tumor) or the thickness of the target tissue (for example, a layer of thin musculoskeletal tissue) is smaller or comparable to the shear wave wavelength, SWS could be affected, a fact that has been reported [22,23].

Engineering factors include the techniques used to evaluate the SWS. These techniques are usually based on a time-of-flight (TOF) approach. In this approach, the times at which the maximum particle displacement of the shear wave is passing at fixed lateral positions are identified. The SWS is calculated as the slope of the line of best fit between these lateral positions and the arrival times. Because these data are noisy, various techniques have been implemented to reduce noise [24] and the SWS is reported as an average and/or median of SWS in the ROI. The SWS value is also affected by the reconstruction method used to determine SWS [25,26]. For commercial ultrasound imaging systems, noise reduction techniques, processing algorithms, and scanner sequencing are proprietary information and are not disclosed by vendors.

Studies have consistently reported a statistically significant difference among the measurements of SWS obtained using ultrasound systems from different vendors (see, for example [27,28,29]). A statistically significant variability in SWS measurements with depth and a probe used has also been reported in the literature [see for example [28,30,31,32,33]]. A prospective clinical study involving 201 patients with nonalcoholic fatty liver disease (NAFLD) showed that both magnetic resonance elastography (MRE) and 2D shear wave elastography (2D-SWE) are highly effective at detecting liver fibrosis. That study also established the optimal liver stiffness thresholds in kPa for each fibrosis stage, based on pathology data from liver biopsy samples. For instance, the optimal threshold for stages 1 and 4 was 2.92 kPa and 4.62 kPa for MRE, and 6.35 kPa and 9.88 kPa for 2D-SWE. These measurements were taken using MRE and SWE systems from the same manufacturer, GE Healthcare [34]. A similar study performed by another group reported a similar trend; the threshold for stages 1 and 4 was 2.6 kPa and 4.6 kPa for MR, and 5.4 kPa and 9.2 kPa for 2D-SWE [35]. Both studies showed that liver stiffness measurements using 2D SWE were twice as high as those obtained with MRE.

Yoo et al. reported that the 2D-SWE thresholds for liver fibrosis were 5.83 kPa for stage 2 and 9.58 kPa for stage 4, using the RS85 (Samsung shear wave imaging) [36]. Additionally, the SWE cutoff values using the Aixplorer (SuperSonic Imagine, Aix-en-Provence, France) were reported as 8.8 kPa for stage 2 and 18.1 kPa for stage 4 [37]. Numerous studies have demonstrated that liver stiffness values measured using different modalities, such as MRE and 2D-SWE, can vary significantly. These values also differ between manufacturers of 2D-SWE systems.

As 2D-SWE is a quantitative imaging modality used for diagnostic and disease staging purposes in clinical settings, it is crucial to ensure that its measurements remain consistent over time, similar to the quality control procedure for advanced imaging modalities like PET, SPECT, CT, and MRI systems. The RSNA Quantitative Imaging Biomarker Alliance (QIBA) SWS biomarker committee has identified biases in SWS measurements and developed a suitable phantom for characterizing data from different systems [38]. Clinical medical physicists routinely conduct acceptance testing and regular quality assurance (QA) tests for ultrasound imaging systems. However, there is currently no regulatory requirement for acceptance testing and quality assurance specifically for SWE, and few publications address this issue from a clinical medical physics perspective [39,40].

Routine QA testing is usually performed using commercially available simple phantoms and does not require the use of computational programs beyond a simple spreadsheet. Each phantom comes with a certificate containing ground-truth values measured by the manufacturer or a calibration lab; however, various groups have demonstrated that SWE measurements can significantly differ from those reported values by the manufacturer [41,42]. Therefore, we believe it is essential to establish baseline SWE measurements using phantoms and to monitor these values to ensure the performance of SWE ultrasound systems. The objective of this study is to develop a quality assurance process that includes establishing baseline data and implementing regular monitoring to ensure constancy and reproducibility.

## 2. Materials and Methods

We used phantoms specifically designed for shear wave elastography. Our medical center is equipped with 38 clinical ultrasound systems from three different manufacturers. To develop a quality assurance procedure, we selected one system from each manufacturer. A set of 3 ultrasound phantoms, Shear wave liver fibrosis phantom (CIRS Inc. Norfolk, VA, USA), consisting of cylinders, each with different stiffnesses labeled as low, medium, and high, were imaged with clinical ultrasound machines from 3 vendors. Several groups measured SWE using the same model of phantoms and found significant discrepancies between the calibrated values provided by the manufacturer and their own measurements [41,42]. Therefore, we measured SWE to establish baseline values for each phantom. We have verified that the weight of each phantom has remained unchanged since the phantom was purchased. Clinical ultrasound machines from three vendors (Philips Epiq, Siemens Sequoia and GE Logiq E10) were used to image the set of three liver fibrosis cylinders using their point shear wave and 2D shear wave applications (Table 1). In the case of one ultrasound machine (GE Logiq E10), only the 2D shear wave was available clinically.

The Triple Modality 3D abdominal phantom (CIRS Inc. Norfolk, VA, USA) is a multi-modality anthropomorphic abdominal phantom capable of being imaged at CT, US, and MR. We used this phantom for ultrasound elastography imaging and compared the results to those from magnetic resonance elastography (MRE) imaging. The liver in the abdominal phantom was manufactured to have a slightly higher stiffness to correspond to early fibrosis. Both the abdominal phantom and the set of 3 cylinders are composed of the same solid hydrogel material with the following properties: density of 1.03 g/cc, speed of sound equivalent to 1540 m/s, and an ultrasound attenuation coefficient of 0.7 dB/cm/MHz.

**Figure 1:**
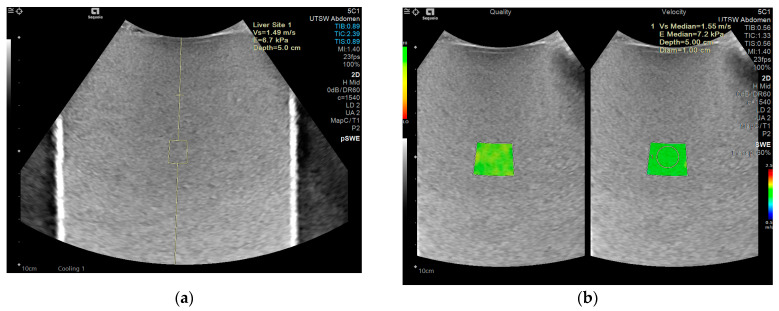
Representative ultrasound elastography images acquired by Siemens (Sequoia) on a medium stiffness cylinder phantom at a depth of 5 cm. The green box indicates a high level of confidence in SWE measurements. (**a**) pSWE image, and (**b**) 2D-SWE image with the confidence map on the left and the elastography 2D-SWE color map on the right.

Phantoms were imaged using the standard ABDOMEN protocol provided on each clinical ultrasound machine. All measurements were conducted in a clinical examination room where the room temperature is regulated to 22 degrees celsius consistently across all Ultrasound rooms. The abdominal probe was positioned freehand, perpendicular to the surface, and near the center of the three cylindrical phantoms, without the use of a probe holder. The anthropomorphic abdominal phantom was imaged such that the largest cross-section of the liver was displayed in B-mode. For each data point presented for the cylindrical phantoms, 10 images were captured, and one ROI was extracted from each image, as shown in Figure 1.

Table 1 lists the probes that were used by each manufacturer along with the selected form of ultrasound elastography, including the results from the MRE.

**Figure 2:**
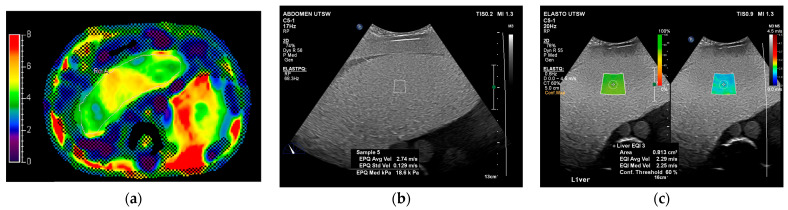
(**a**) An axial view of MR elastography with a goodness-of-fit confidence overlay (Philips Insignia 1.5 T). Sagittal views of ultrasound elastography: (**b**) pSWE and (**c**) 2D-SWE ROIs (Philips Epiq), both acquired from the same anthropomorphic abdominal phantom.

A good-quality B-mode image is essential for accurate SWE measurements. The B-mode image guides the placement of the ROI within a homogeneous area of the liver parenchyma, avoiding vessels and bile ducts. Since an ROI box is usually placed in the middle center of the B-mode image, it is recommended to position the ROI away from the liver dome, ribs, and lungs to prevent refraction artifacts. The B-mode image shown in Figure 2 was obtained using 78% of 2D gain for overall brightness, a dynamic range of 55 gray levels, medium persistence for frame averaging, and Gen for 2D optimization to balance resolution and penetration. Similar parameters for abdomen imaging were used for the other two manufacturers. Once the B-mode image was optimized, the elastography function was engaged, first by using point shear wave and subsequently using 2D shear wave, which displays side-by-side images of the confidence map with a color map representing a continuum of shear wave velocities present in the region of interest, as shown in Figure 2.

Vendors recommend positioning the ROI on a uniformly color-coded region with a confidence level greater than the preset threshold. The confidence level in 2D-SWE reflects the quality of the measured stiffness value in each pixel within a region of interest (ROI) box. All manufacturers provide a confidence map with a color-scale bar ranging from 0 to 100%, where 100% indicates the highest shear wave strength. A confidence threshold can be applied to the fused view of B-mode and 2D-SWE data. When the confidence level of pixels falls below this threshold, the corresponding SWE values are deemed unreliable and inconsistent. Philips employs a 60% confidence threshold specifically for liver stiffness measurements, clearly displaying this value on the acquisition screen alongside a confidence scale ranging from 60% to 100%. GE utilizes a confidence scale from 0% to 100%, highlighting a recommended confidence threshold of 45%. In contrast, Siemens uses a confidence scale without explicit indication of the confidence threshold, categorizing it as low to high.

Our clinical default settings for ultrasound systems vary according to clinical needs. On GE and Siemens systems, the measured elastography values are displayed as Young’s modulus in kPa. However, the Philips systems display shear wave speed (SWS) in 2D-SWE, which can be converted to a Young’s modulus, as indicated in Equation (1).

In order to validate the measurements between the phantoms and to establish the baseline value, the abdominal phantom was imaged with MR elastography as it is accepted as a non-invasive standard for staging liver fibrosis [43,44]. The MR scanner used in this study was the Philips Ingenia 1.5 T with the Resoundant MR Elastography System (Resoundant, Rochester, MN, USA), which was designed for non-invasive assessments of liver stiffness. The passive driver was placed on top of the liver in the phantom and then firmly secured using the belt provided by Resoundant. The phantom bundle was then positioned in the middle of a torso phased-array coil. The same driver frequency of 60 Hz as used in the in vivo clinical study was used here. The clinical imaging protocol was performed to generate MR elastograms from 4 axial slices (slice thickness = 10 mm) through the liver in the phantom.

## 3. Results

Our initial plan was to measure SWE within the 1–7 cm depth range. However, all systems reported unreliable SWE values, particularly beyond 6 cm and near the phantom’s entrance, making data collection at these locations impossible. Consequently, we presented all measurable data, primarily focusing on depths ranging between 3 and 6 cm. Manufacturers recommend acquiring SWE measurements at a depth of 3–6 cm, with optimal quality typically achieved at a depth of around 4–5 cm.

Figure 2 is representative of the ultrasound image alongside the equivalent MRE at a depth of 5 cm. We followed a standard clinical procedure for liver stiffness measurement, involving an assessment of the entire liver rather than using a small ROI, and calculation of the average MRE values within the liver contours. Similarly, the average SWE was determined by averaging measured values across depths ranging from 4 to 6 cm. A scaling factor was obtained between the two in order to substantiate acquired measurements. We summarized the average MRE, pSWE, and 2D-SWE from each US system in Table 2.

MRE measures the magnitude of the complex shear modulus (*G*), whereas ultrasound measures Young’s modulus (*E*), as an indicator of stiffness. In isotropic materials, these moduli are related by the equation *E* = 2*G*(1 + ν), where ν represents Poisson’s ratio. For soft tissues like the liver, ν is about 0.5, yielding a scaling factor, *E/G*, of approximately 3. This aligns closely with the scaling factors observed in our experiments, particularly with the Siemens ultrasound system.

Since the MRE value is considered to be the non-invasive standard for elastography, the scaling factor calculated above indicates that the abdomen phantom under ultrasound elastography is measuring higher by a factor of 4 in pSWE and 3.39 in 2D-SWE for the same manufacturer. We do not have the corresponding data to make any statement regarding the clinical use of elastography and any scaling factor between MRE and ultrasound elastography from any other manufacturer. The difference between pSWE and 2D-SWE was significant for the Philips system (*p* < 0.05), while it was not significant for the Siemens system. The GE system did not include a pSWE feature. The percent coefficient of variation of pSWE and 2D-SWE across three depth positions (4–6 cm) was less than 5% for both the Philips and Siemens systems, while it was 13% for GE’s pSWE measurements.

**Figure 3:**
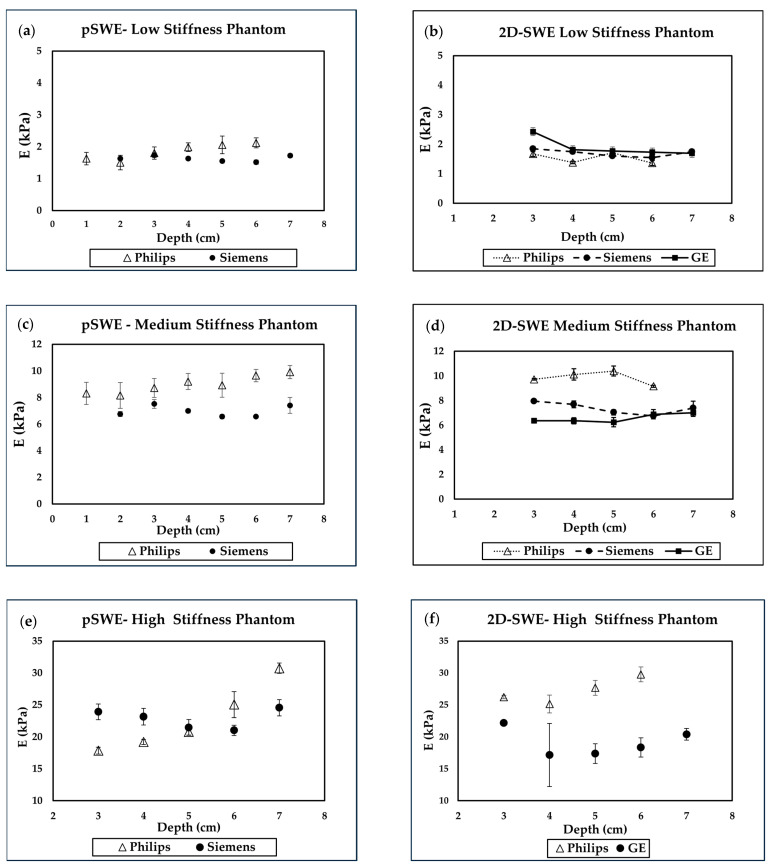
Depth dependence of pSWE and 2D-SWE for the low- and medium-stiffness phantoms. All average and standard deviation values are derived from three or more measurements. Note that small standard deviation values may not be clearly visible. (**a**) pSWE of the low-stiffness phantom using Philips and Siemens systems; (**b**) 2D-SWE of the low-stiffness phantom using the Philips, Siemens, and GE systems; (**c**) pSWE of the medium-stiffness phantom using the Philips and Siemens systems; (**d**) 2D-SWE of the medium-stiffness phantom using the Philips, Siemens, and GE systems; (**e**) pSWE for the high-stiffness phantom; and (**f**) 2D-SWE of the high-stiffness phantom using the GE system.

Figure 3 shows the data from pSWE and 2D-SWE for the low-, medium-, and high-stiffness cylindrical phantoms. The 2D-SWE values of the high-stiffness phantom were too high for the liver protocol we utilized. Consequently, due to low confidence in 2D-SWE measurements by the Siemens system, we only reported 2D-SWE results for the GE and Philips systems. The paired t-test indicated significant differences between pSWE and 2D-SWE measurements for the low-stiffness phantom in both the Philips (*p* < 0.05) and Siemens systems (*p* < 0.05). However, significant differences were observed only in the Siemens system (*p* = 0.01) for the medium-stiffness phantom. pSWE data from Philips showed significantly higher values compared to Siemens in both the low-stiffness (*p* = 0.02) and medium-stiffness (*p* = 0.02) phantoms. For 2D-SWE measurements, significant differences were observed among all three systems in the low-stiffness phantom, whereas the difference was small in the medium-stiffness phantom.

From the abdominal phantom, one set of measurements using 2D shear wave elastography was made at the same depth but at different lateral locations while still well positioned within the liver. When the B-mode image is acquired, an ROI box for SWE measurements is automatically placed in the center. However, during clinical exams, the sonographer or clinician can move the box laterally or to a different depth within the liver parenchyma to assess overall tissue characteristics. Therefore, we investigated the variability of SWE measurements at three lateral locations—left, center, and right—within the B-mode image while avoiding the liver boundary. Table 3 is a summary of the shear stress measurements at 6 cm acquired from the three ultrasound manufacturers on the multi-modality anthropomorphic abdomen phantom. The average percent deviation relative to the center measurement was highest for the Philips system.

**Figure 4:**
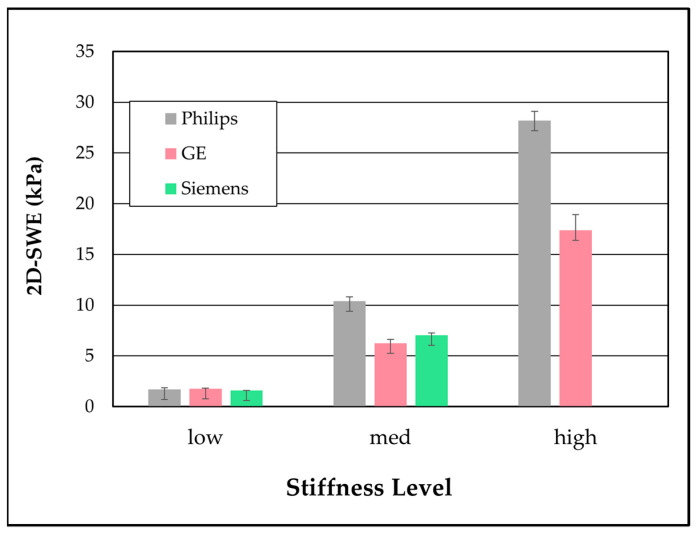
Two-dimensional shear wave elastography measurements acquired at 5 cm below the surface of the three-cylinder phantoms. Each data point represents the average and standard deviation of ten measurements.

In Figure 4, the data from 2D SWE acquired using each system at 5 cm were compared based on their phantom stiffness. It is notable that for the low-stiffness cylinder, all three manufacturers measured almost the same value, 1.55, 1.60, and 1.77 kPa for GE, Siemens, and Philips, respectively; however, as the stiffness value increases, the measurements diverge such that for the high-stiffness cylinder phantom, there is a substantial difference (17.4 for GE and 28.2 kPa for Philips).

**Figure 5:**
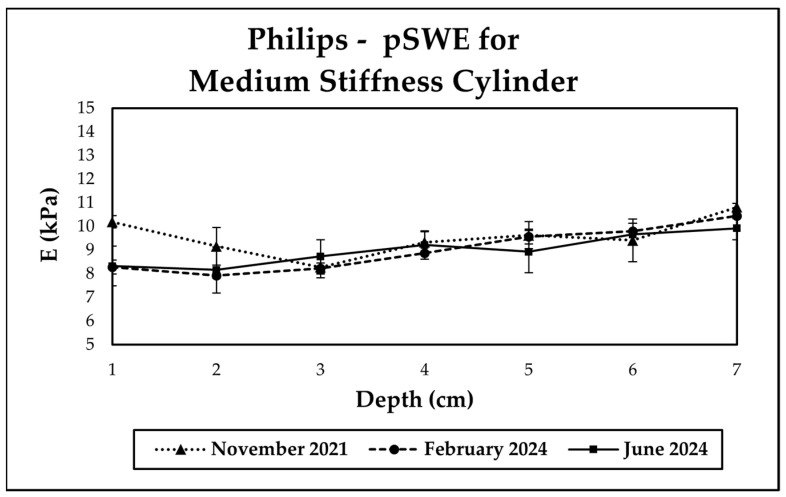
Repeatability of point shear elastography (pSWE) measurements. The data plotted represent the average value of ten measurements and standard deviation.

We also checked that our data were repeatable over the years, though data were not acquired every year. Figure 5 illustrates the case of the Philips Epiq pSWE repeatability for a time interval of 3 years between measurements. Philips does mention in their instructions for elastography that measurements should be taken 3 cm below the liver capsule; hence, the data at 1 and 2 cm are not representative of the vendor’s instructions. The maximum coefficient of variation for pSWE measurements within the 3–6 cm depth range over approximately three years was 4%.

## 4. Discussion

Phantom measurements are not necessarily clinical indicators but do show trends that can be followed during subsequent testing. It is also valuable to check that MRE and US scanners can be cross-calibrated by the medical physicist to ensure that patients who are imaged by both methods have the best treatment or patient management plan. It is worth performing a retrospective of patients who were imaged with both modalities within a short period between measurements to track MRE vs. US elastography data. The baseline MRE value can be also used for future root-cause analyses of US systems. If SWE measurements deviate significantly from the baseline, MRE will be acquired again to investigate the cause of the changes. If MRE remains constant while SWE changes, it will be determined that the changes are due to factors other than the phantom, such as equipment or software-related issues.

Sogrist’s group performed an intra-individual comparison between two elastography systems to investigate whether these systems can be used interchangeably to grade liver fibrosis [45]. The average SWS for GE was significantly lower than the average SWS measurements obtained with the Siemens system. Their results suggest that quantitative SWE cannot be used interchangeably to grade liver fibrosis in the same patient longitudinally. Therefore, for patients with subsequent ultrasound visits, it is important to ensure that all elastographies are imaged on the same scanner. That is, a patient who is first imaged on a Siemens should have all their subsequent measurements on a Siemens and not on another platform. There is some variability between manufacturers due to their unique implementation of the algorithms forming the elastography function.

The technical standard developed jointly by the American College of Radiology (ACR), the American Association of Physicists in Medicine (AAPM), and the Society for Imaging Informatics in Medicine (SIIM) recommends that physicians and qualified medical physicists actively participate in the QA procedures to ensure that the image meets the necessary standards for accurate diagnosis [46]. Hangiandreou’s group found that the annual failure rates for scanner components and transducers were 10.5% and 13.9%, respectively [47]. Their study also emphasized that a simple QA test such as a uniformity evaluation could detect 66.3% of all failures, highlighting the critical role of QA in using ultrasound for patient care. The ACR and AAPM collaboratively developed a technical standard for ultrasound equipment [48]. Acceptance testing of the scanner and transducers should include physical and mechanical inspection, transducer port inspection, image uniformity and artifact survey, system sensitivity, spatial resolution, contrast resolution, near-field assessment, and fidelity of ultrasound scanner electronic image display. For systems with advanced capabilities such as Doppler or elastography, additional tests should be performed. Furthermore, some of these tests are repeated annually as part of the QA program. However, the technical standard does not specify how each test should be conducted; therefore, each institution establishes its own QA procedure to comply with the standard.

It would also be desirable that phantom tissue-mimicking materials be more directly correlated to human tissue. As a method to track functionality though, it is only necessary to select one phantom and measure it against all vendors at specific depths to have an estimate of whether measurements are still valid, irrespective of phantom material. If MRE would also be available, then correlations between manufacturers could be implemented, as well, to determine the scaling factors between MRE, US elastography, and the various manufacturers during acceptance testing, thereby providing the benchmark for future testing and measurements. We have shown the 3-year repeatability for the Philips system. A similar study should be conducted for other systems when implementing a QA program.

## 5. Conclusions

Our study demonstrates that discrepancies exist when evaluating the stiffness of liver fibrosis using phantoms on ultrasound systems from three different vendors. The pSWE and 2D-SWE measurements taken on the same system can show significant variations. This discrepancy is more evident in materials with high stiffness compared to those with low stiffness. The precision of Young’s modulus in a 1 cm circular ROI at a depth of 5 cm was found to be 5% for the GE, 3% for the Siemens, and 6% for the Philips systems on a medium stiffness phantom. Considering the 4% repeatability of elastography measurements for the Philips system over time and the precision measurements, the acceptable percent difference between baseline and subsequent measurements should be within ±10%. We have shown that it is feasible to assess the consistency of elastography in clinical systems by incorporating phantoms into routine quality assurance procedures. Pass and fail criteria can be established based on each system and institutional requirements.

## Figures and Tables

**Table 1:** List of imaging equipment, probes selected for elastography, and acquisition modes for the phantom study.

Equipment (Model)	Software Version	Probe	Acquisition	Anthropomorphic Abdominal Phantom	Shear Wave Liver Phantoms
GE (Logiq E10)	R3	C1-6	2D shear wave	x	x
Philips (Epic 5G)	9.0	C5-1	Point and 2D shear wave	x	x
Siemens (Sequoia)	VA40A	5C1	Point and 2D shear wave	x	x
Philips 1.5 T (Ingenia MRE)	5.1.7.3			x	

**Table 2:** Comparison of average MRE and US elastography measurements over an optimal depth range (4–6 cm). Measurements were obtained from MRE and three ultrasound manufacturers using an anthropomorphic abdomen phantom.

Image Modality	Young’s Modulus (kPa)	Shear Modulus (kPa)	Scaling Factor
**MRE**			
Philips Insignia 1.5 T		4.54 ± 0.01	
**Ultrasound**			
Philips Epic 5G			
pSWE	18.43 ± 0.31		4.06
2D-SWE	15.35 ± 0.33		3.38
Siemens (Sequoia)			
pSWE	13.61 ± 0.61		3
2D-SWE	13.58 ± 0.62		3
GE (Logiq E10)			
2D-SWE	11.90 ± 1.53		2.62

**Table 3:** Lateral variability of 2D SWE within the abdomen liver phantom at 6 cm from the surface.

Equipment	Left (kPa)	Center (kPa)	Right (kPa)	Mean (kPa)	%Deviation (Mean-Center)/Center
GE	14.5	13.3	14.3	14.1	4%
Philips	16.6	14.7	18.5	16.6	13%
Siemens	13.5	14.1	12.8	13.5	4%

## Data Availability

The authors will provide the raw data supporting the conclusions of this article upon request.
